# 17-Month-Old Girl With Severe, Prolonged Lethargy and Somnolence

**DOI:** 10.7759/cureus.16807

**Published:** 2021-08-01

**Authors:** Allison C Lure, John-Anthony Coppola, Freddie R Guyer, Avni Bhatt

**Affiliations:** 1 Department of Pediatrics, University of Florida, Gainesville, USA

**Keywords:** adenovirus, encephalopathy, lethargy, cornell assessment for pediatric delirium

## Abstract

A 17-month-old girl arrived at the pediatric ED with decreased responsiveness. She was lethargic, localizing only to noxious stimuli with vital signs significant for fever of 103.8 °F, heart rate of 185 beats/min, respiratory rate of 12 breaths/min, blood pressure of 100/59 mmHg, and oxygen saturation level of 88% on room air. She was admitted to the pediatric intensive care unit (PICU) due to concerns of septic meningitis with altered mental status and respiratory distress, and was treated with antibiotics. A respiratory viral panel (RVP) was positive for adenovirus, resulting in all antibiotics being discontinued. She remained lethargic until day nine of illness, when she had improved almost completely to her baseline. Polymerase chain reaction (PCR) of her cerebral spinal fluid returned positive for adenovirus serotype A, thus confirming our case of transient adenovirus encephalopathy. This case illustrates the importance of keeping adenovirus in the differential for encephalopathy versus a neurologic abnormality or other malignant or infectious etiology.

## Introduction

Human adenovirus is a double-stranded DNA virus with over 60 serotypes divided into seven subgroups, or species [1]. The virus triggers both the innate and adaptive immune response in humans, and its various clinical presentations depend on age group, immune status, and adenovirus species [2]. Most infections typically cause mild self-limiting symptoms, primarily in children, ranging from respiratory, GI, urinary, and keratoconjunctivitis [3]. Rarely do they cause more severe symptoms such as hepatitis, pancreatitis, nephritis, encephalitis, hemorrhagic cystitis, or hemorrhagic colitis [3]. The clinical presentation can be more severe in patients who are immunocompromised, especially those with defects in cell-mediated immunity [2-4]. Here we report a unique case of adenovirus-related encephalopathy in a healthy child without impaired immunity.

## Case presentation

A 17-month-old girl with a past medical history of reactive airway disease, up to date on her vaccinations, presented to the ED via ambulance due to decreased responsiveness. Her mother reported that she began having nasal congestion, cough, and fever with a maximum temperature of 103°F three days prior to presentation. She was taken to an outside facility at this time and diagnosed with an acute otitis media and started on cefdinir. One day prior to presentation, she had increased work of breathing, decreased oral intake, decreased urine output, and two episodes of non-bloody, non-bilious emesis.

In the ED, the physical exam showed vital signs significant for fever of 103.8 °F, heart rate of 185 beats/minute, respiratory rate of 12 breaths/min, blood pressure of 100/59 mmHg, and oxygen saturation of 88% on room air. She was lethargic, localizing only to noxious stimuli. She had copious yellow nasal discharge, a bulging right tympanic membrane, dry mucous membranes, and anterior cervical lymphadenopathy. Her bradypnea and mental status both improved after suctioning. Her lungs were clear to auscultation with no wheezing or rhonchi. She had a 2/6 systolic murmur in the left sternal border and a capillary refill of five seconds with warm extremities. The remainder of her exam was normal. She was on day three of a 10-day course of cefdinir 14 mg/kg/day, administered every 12 hours.

A CT scan of her head showed no acute intracranial abnormality. Initial laboratory evaluation showed respiratory alkalosis with a venous pH of 7.48, and pCO2 of 37 mmHg. A complete blood count with differential was normal. She had an elevated C-reactive protein of 93.41 mg/L. Her complete metabolic panel showed hyponatremia with a value of 134 mmol/L and hypochloremia with a value of 94 mmol/L. Her chest X-ray, ECG, and urinalysis were all unremarkable. Her blood and urine cultures were negative. A lumbar puncture was performed, and her cerebrospinal fluid (CSF) had a normal cell count with WBC of 2 cu mm, glucose of 88 mg/dL, and protein of 17 mg/dL. Enterovirus polymerase chain reaction (PCR) of the CSF was negative. Herpes simplex virus PCR was not done. The CSF cultures were negative. A rapid respiratory syncytial virus (RSV) test was found to be positive.

Due to her lethargy, there was a concern for accidental ingestion and she was given naloxone 0.1 mg/kg immediately on the presentation without improvement of symptoms. However, because she was slightly more alert after nasal suctioning, and history revealed multiple days of fever, an infectious etiology was deemed the most probable cause and no toxicology workup was completed. She received a 20 ml/kg bolus of normal saline and was then started on maintenance intravenous fluids. Due to concern for meningitis, she was started on ceftriaxone 100 mg/kg daily and vancomycin 15 mg/kg/dose every six hours after CSF was obtained. She was placed on a high-flow nasal cannula and admitted to the pediatric intensive care unit (PICU) for respiratory distress, altered mental status, and concern for meningitis.

In the PICU, she remained febrile but hemodynamically stable. Vancomycin was discontinued on hospital day one, and she was weaned to room air, resulting in a transfer to the general pediatric inpatient service. Due to her persistent fever, a nasopharyngeal respiratory viral panel (RVP) was performed. When the RVP returned positive for adenovirus, and the cultures remained negative for 48 hours, ceftriaxone was discontinued. No MRI, EEG, or repeat lumbar puncture was deemed necessary. While on the inpatient service, the patient remained lethargic, barely reaching for her bottle. Each day her symptoms improved, and she eventually returned close to her baseline on days five and six of hospitalization (Table 1). Because adenovirus infection can present in a variety of ways [3], adenovirus PCR of the CSF was sent and returned positive for adenovirus serotype A, which confirmed the diagnosis of adenovirus encephalopathy.

**Table 1:** Cornell Assessment of Pediatric Delirium score during hospitalization.

	Day 1 (Presentation Day)	Day 2	Day 3	Day 4	Day 5	Day 6 (Discharge Day)
1: Eye contact			4	4	0	0
2: Purposeful actions			3	3	2	0
3: Awareness of surroundings			4	4	2	0
4: Communicate needs and wants			4	4	2	0
5: Restless			4	3	3	2
6: Inconsolable			4	4	3	1
7: Underactivity			4	4	3	2
8: Response times to interactions			4	4	3	1
Total score			31	30	18	6

The patient was discharged on day six of hospitalization with a final diagnosis of adenovirus encephalopathy and bronchiolitis. Given her clinical improvement and no known history of immunocompromise, she was not treated with antiviral therapy and instead was given supportive care.

## Discussion

This patient’s initial presentation to the ED was concerning due to her lethargy and respiratory distress. In a 17-month-old girl, the differential diagnosis is vast. Ingestion is high on the differential, however, in this patient, there was no known opportunity and naloxone was ineffective in reversing her symptoms. Her high fever favored an infectious cause and no toxicology workup was pursued. While bacterial meningitis was a concern, her lumbar puncture was reassuring. She was fully vaccinated and she had no exposure to sick contacts. Her positive RSV and known resolving acute otitis media in part explained her fever and nasal congestion, however, the duration and severity of lethargy (Table 1) were atypical for these illnesses. The positive RVP for adenovirus guided us to the final diagnosis of adenovirus encephalopathy which was confirmed via PCR of her CSF.

Adenovirus infection can be diagnosed through viral culture, viral antigen assay, PCR, histopathology, and serology [5]. Serotyping and genotyping can be done to further identify the species [5].

Recovery from adenovirus infection is associated with the development of serotype-specific neutralizing antibodies towards the major capsid protein. Neutralizing antibodies protect against infection with the same serotype of the virus but not against other serotypes [6]. While adenovirus can cause fatal systemic disease in immunocompromised patients, our patient was not known to be immunocompromised. While she showed systemic symptoms, she also improved on supportive care alone, and therefore antivirals were not started. There are currently no controlled trials that have demonstrated benefit in the use of antivirals for adenovirus, however, they are still used in severe cases. Cidofovir and ribavirin have been shown through clinical trials to have efficacy against adenovirus [3].

Adenovirus encephalopathy has only been reported in a handful of cases [7-10]. It was first reported in 1956 in France, by Chany et al. where adenovirus was isolated from CSF fluid [10]. In 1978, Kelsey describes four cases of children ages four through ten, who presented with adenovirus positive CSF [11]. In these cases, the CSF studies were abnormal with elevated leukocyte counts [11]. This is in contrast to this patient whose CSF studies were normal with a WBC of 2 cu mm. In 2001, Straussberg et al. reported on seven patients with a decreased level of consciousness and positive adenovirus infections from various samples, although notably not from the CSF [9]. In 2014, Reyes-Andrade et al. reported a case of meningoencephalitis in a healthy 15-month-old who presented with similar, albeit more severe, symptoms than this patient [7]. A retrospective review was completed by Lema et al. who evaluated 108 CSF samples from a combination of encephalitis, meningitis, and other neurologic patients and found 5.5% of the cases were positive for adenovirus, all being from the encephalitic patient cohort [12]. A retrospective study conducted by Huang et al. identified CNS involvement, most notably seizures, in 3.3% (109/3298) of the cases of culture-confirmed (throat or nasopharyngeal swab) adenovirus infections [8]. Of the patients with CNS involvement, 62% had seizures, 13% had altered mental status, and 8% had lethargy [8]. These retrospective studies established the presence of neurological sequelae in adenovirus infection. Furthermore, a 2018 study of immunocompetent children with adenovirus-associated CNS disease demonstrated the range of neurologic complications from fully reversible encephalopathy to fatal, acute necrotizing encephalopathy [13]. In their study, 38% (18/48) of the infections were fatal or resulted in permanent neurologic sequelae [13].

## Conclusions

Although adenovirus is a common pediatric viral illness, it is usually associated with mild localized illness, and the reporting of adenovirus encephalopathy is rare. In the diagnosis of encephalopathy, it is important to consider that adenovirus can present with a high degree of altered mental status without a focal neurologic abnormality. We hope this case helps support the literature in understanding the role of adenovirus in patients with encephalopathy.

## References

[1] Benfield DA, Hesse RA (2019). Adenoviruses. In: Diseases of Swine.

[2] Khanal S, Ghimire P, Dhamoon AS (2018). The repertoire of adenovirus in human disease: the innocuous to the deadly. Biomedicines.

[3] Lynch JP 3rd, Kajon AE (2016). Adenovirus: epidemiology, global spread of novel serotypes, and advances in treatment and prevention. Semin Respir Crit Care Med.

[4] Kojaoghlanian T, Flomenberg P, Horwitz MS (2003). The impact of adenovirus infection on the immunocompromised host. Rev Med Virol.

[5] Langley JM (2005). Adenoviruses. Pediatr Rev.

[6] Zaiss AK, Machado HB, Herschman HR (2009). The influence of innate and pre-existing immunity on adenovirus therapy. J Cell Biochem.

[7] Reyes-Andrade J, Sánchez-Céspedes J, Olbrich P (2014). Meningoencephalitis due to adenovirus in a healthy infant mimicking severe bacterial sepsis. Pediatr Infect Dis J.

[8] Huang Y-C, Huang S-L, Chen S-P, Huang Y-L, Huang C-G, Tsao K-C, Lin T-Y (2013). Adenovirus infection associated with central nervous system dysfunction in children. J Clin Virol.

[9] Straussberg R, Harel L, Levy Y, Amir J (2001). A syndrome of transient encephalopathy associated with adenovirus infection. Pediatrics.

[10] Chany C, Lépine P, Lelong M, Le-Tan-Vinh Le-Tan-Vinh, Satgé P, Virat J (1958). Severe and fatal pneumonia in infants and young children associated with adenovirus infections. Am J Epidemiol.

[11] Kelsey DS (1978). Adenovirus meningoencephalitis. Pediatrics.

[12] Lema CL, Cisterna DM, Freire MC (2005). [Neurologic disease due to adenovirus infection]. Medicina (B Aires).

[13] Schwartz KL, Richardson SE, MacGregor D, Mahant S, Raghuram K, Bitnun A (2019). Adenovirus-associated central nervous system disease in children. J Pediatr.

